# The Effect of Clearance Angle on Tool Life, Cutting Forces, Surface Roughness, and Delamination during Carbon-Fiber-Reinforced Plastic Milling

**DOI:** 10.3390/ma16145002

**Published:** 2023-07-14

**Authors:** Tomáš Knápek, Štěpánka Dvořáčková, Martin Váňa

**Affiliations:** Assembly and Engineering Metrology, Department of Machining, Faculty of Mechanical Engineering, Technical University of Liberec, 461 17 Liberec, Czech Republic

**Keywords:** milling, CFRP, tool wear, tool parameters, delamination

## Abstract

This study aimed to investigate the effect of the clearance angle of the milling tool on wear, cutting forces, machined edge roughness, and delamination during non-contiguous milling of carbon-fiber-reinforced plastic (CFRP) composite panels with a twill weave and 90° fiber orientation. To achieve the objective of the study, it was first necessary to design suitable tools (6 mm diameter sintered carbide shank milling cutters) with a variety of clearance angles (8.4°, 12.4°, and 16.4°) and all the machinery and measuring equipment for the research to be carried out. Furthermore, measurement and evaluation methods for cutting tool wear, cutting forces, machined edge roughness, and delamination were developed. Last but not least, the results obtained during the research were summarized and evaluated. From the experiments conducted in this study, it was found that the tool clearance angle has a significant effect on tool wear, roughness of the machined surface, and delamination of the carbon fiber composite board. The tool with a clearance angle of 8.4° wore faster than the tool with a clearance angle of 16.4°. The same trend was observed for cutting force, machined surface roughness, and delamination. In this context, it was also shown that the cutting force increased as the tool wear increased, which in turn increased surface roughness and delamination. These results are of practical significance, not only in terms of the quality of the machined surface but also in terms of time, cost, and energy savings when machining CFRP composite materials.

## 1. Introduction

The machining of composite materials is highly desirable due to their widespread use in various industries. However, machining these composite materials poses several difficulties due to their properties, which differ significantly from those of traditional metallic materials [1,2].

Key parameters that affect the cutting process include cutting conditions, such as axial and radial depth of cut, engagement angle, feed, and feed rate profile. These parameters, along with their variations along the tool path, have a direct impact on cutting speed, tool wear, and cutting force [3,4,5].

The damage forms and failure modes of carbon-fiber-reinforced plastic (CFRP) materials are strongly influenced by changes in their microstructures and interface properties. These properties include fiber orientation, fiber and matrix volume fraction, as well as the presence of voids and cracks. Since CFRP composites consist of a reinforced phase (carbon fiber) and a continuous phase (epoxy resin), the machining process for CFRP is more complex compared to that of homogeneous materials [6,7].

When machining CFRP, it is recommended not to exceed the glass transition temperature (Tg) of the material matrix. The cutting temperature significantly influences the subsurface damage of the hole wall, thereby influencing the tensile performance of the material [8,9].

The direction of the CFRP fiber has a significant effect on the quality of the machined layer and the wear of the cutting tool. The mechanical interaction between CFRP and the cutter is primarily influenced by the anisotropy of the material and the angle of the cutter. In CFRP, the fibers bear the load while the resin facilitates force transfer between the fibers. Therefore, the failure modes of the material can be analyzed primarily based on the stresses experienced by the fibers [10,11].

During milling in a direction parallel to the fiber direction (0°), the tool moves at a specific speed, and the main cutting force is generated by the interaction between the tool face and the material. This cutting force can be divided into components that are parallel and perpendicular to the fiber direction. The perpendicular force generates compressive stress on the fibers, causing them to be pushed and bent (buckling stress). Cracks formed in the fibers at the points of tensile stress lead to fiber breakage. The brittle polymer matrix surrounding the fibers is also subjected to compression, resulting in cracks and fragmentation. The direction of deformation caused by buckling and the location of cracks are primarily influenced by the tool angle [12,13,14].

**Figure 1:**
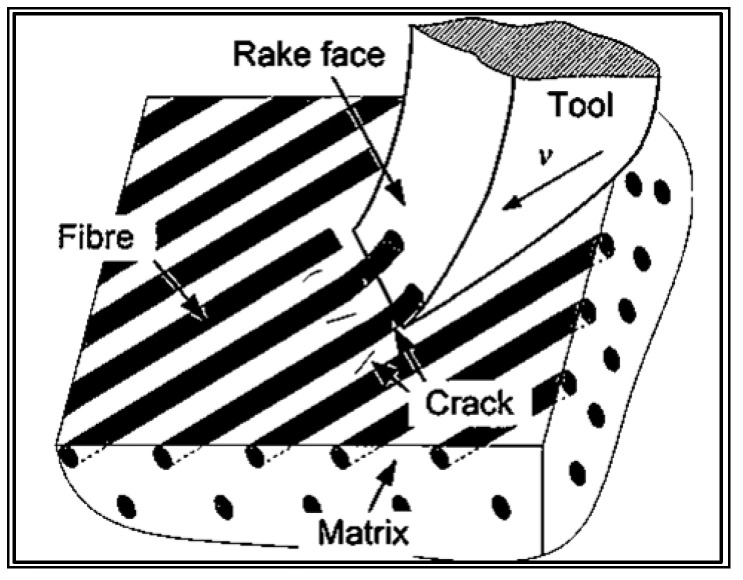
CFRP material removal mechanism at 0° fiber direction [12].

Figure 1 illustrates the material removal mechanism for carbon-fiber-reinforced polymer composites machined in a direction parallel to the fiber direction (0°).

When machined in the direction of the fibers at 90°, the primary cutting force acts perpendicular to the fiber axis and can be divided into cutting forces within the fiber plane and those perpendicular to it. The fibers experience compressive stress at the point of contact with the tool, which can lead to crack formation. On the opposite side of the fiber, tensile stress occurs, which causes fiber breakage. The polymer matrix undergoes compressive stress in front of the cutter, resulting in crack formation and fragmentation of the brittle matrix into small particles [12,15,16,17].

**Figure 2:**
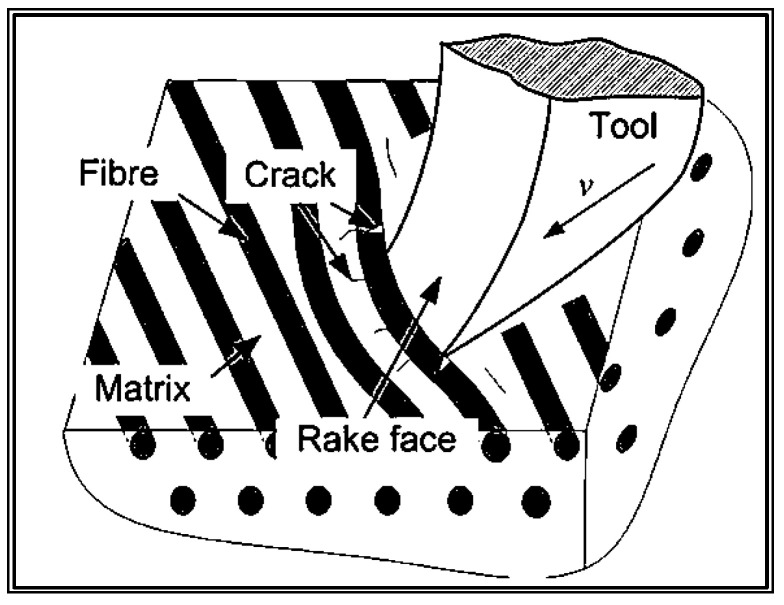
CFRP material removal mechanism at 90° fiber direction [1].

Figure 2 illustrates the material removal mechanism of CFRP when machining at a fiber angle of 90°.

The completely different mechanical parameters of the reinforcing fibers and the matrix used are reflected in the machining in terms of the quality of the machined surface by the so-called delamination. Delamination, which is the most problematic, occurs because it exceeds the forces that hold the composite layers together. Therefore, it is determined by the cohesion of the individual layers of the composite applied, and also by the strength of the bond between the fibers and their binder. According to the literature [18,19,20], delamination can be described as follows. 

During machining, the tool penetrates the composite material. Under pressure, the reinforcing fibers bend, leading to the formation of tensile cracks at the top of the fibers. The resulting cracks initiate the breakage of the fibers. On the bottom side, the fibers are pushed into the resin. The resin beneath the fibers is compressed and crushed. As the fibers in the upper layer of the machined specimen break, they become dislodged when the tool penetrates the material. Consequently, the upper layer of the material lacks fibers. This type of delamination is commonly referred to as type I delamination. Type I delamination is frequently observed when machining in the direction of the fibers at 45° and 90° [21].

The tool applies pressure to the reinforcing fibers, and the fibers bend or bend away from the path of the moving tool. The bending or avoidance of the fibers causes them to be uncut, which then return to their original position in the composite material. This results in protruding fibers on the machined surface. This is type II delamination. Type II delamination occurs most commonly when machining in the direction of the fibers at an angle of 135° [22,23].

**Figure 3:**
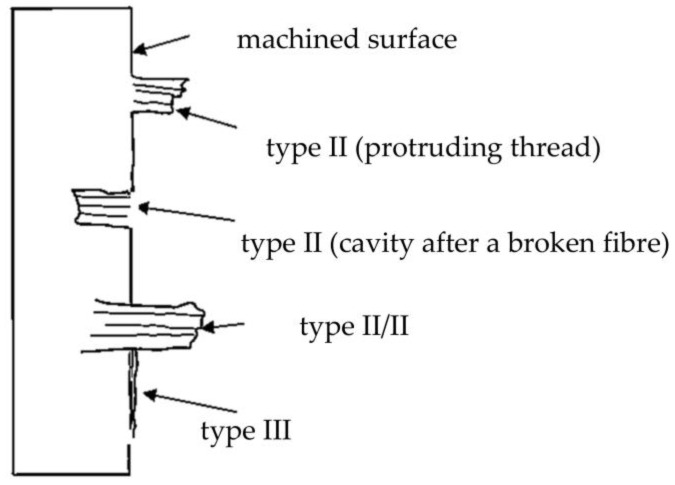
Types of delamination during milling [8].

Type III delamination describes loose fibers that partially adhere to the machined surface and lie parallel to the tool feed direction. Both Type I and Type III delaminations generate loose fibers “attached” to the machined surface and cause a poor quality “unsharp” machined surface very reminiscent of the burrs known from machining metallic materials. The types of delamination are shown in Figure 3 [24].

In recent years, numerous studies have been conducted to address the issue of delamination in machining composite materials, with the aim of developing phenomenological and empirical models to predict its occurrence and emergence. These models serve as valuable tools for implementing delamination as a process monitoring criterion.

Hintze et al. [25] studied the occurrence and propagation of delamination and fiber protrusion during circumferential milling of unidirectional carbon fiber composite material (hereafter referred to as CFRP) and reported that delamination is closely related to the cutting edge (blade) wear and fiber orientation of the upper layer of the composite material. The occurrence of delamination was found to be more frequent in cutting tools with large tooltip radii and when machining composite materials with 90° and 180° fiber orientation. Subsequently, an analytical model was later derived to predict the length of protruding filaments [21]. A model for the analysis of the protrusions was developed by Hosokawa et al. [26], who investigated the effect of the helix angle of a shank cutter on tool wear and delamination during edge trimming of a multidirectional CFRP laminate. It was shown that machining with a large helix angle tool resulted in a smoother surface and less tool edge wear. In addition, tool wear and delamination were reduced when the milling direction was tilted so that the direction of the resultant force was parallel to the feed direction. This was subsequently confirmed by Qingliang et al. [19], who showed that minimal delamination damage was achieved when the tilt angle was equal to the helix angle of the cutter. They also showed that the type of delamination and its frequency of occurrence depended on the inclination angle and fiber orientation. When the inclination angle of the fibers was large, type I/II was the prominent type of delamination. At a low inclination angle, type I delamination occurred more frequently at 45° and 90° fiber orientations, and type II delamination occurred more frequently at 135° fiber orientation [27,28].

Despite the widespread use of composite materials reinforced with more than just carbon fiber, very few studies have attempted to systematically characterize the size and amount of burrs in CFRP machining to determine tool life, mainly due to the complexity of the geometry.

Therefore, the present study focuses on tool wear as a function of tool geometry and fiber orientation, and the analysis of the effect of tool wear on the resulting delamination in CFRP milling.

## 2. Materials and Methods

A laminated 3K CFRP board with a thickness of 4.3 mm was chosen as the machined material. The board was manufactured using the vacuum infusion method. An LG 120 epoxy resin (GRM Systems, Olomouc, Czech Republic) with an HG 356 hardener (GRM Systems, Olomouc, Czech Republic) was used as the matrix. For reinforcement, a CCH600 fabric (Kordkarbon, Strážnice, Czech Republic) with a weight of 600 g/cm^2^ and a twill weave of 2 × 2 cm was used. The surface of the laminate consisted of a thin layer of pure resin with a thickness of 10–15 µm. The properties of the machined materials are shown in Table 1.

**Figure 4:**
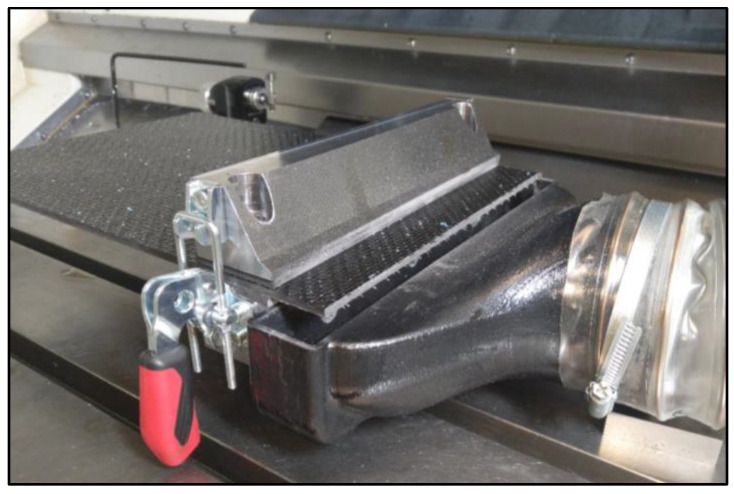
Clamping fixture with a suction device.

The plates used in this study had dimensions of 600 × 250. The choice of 250 mm width dimension was based on the design of a fixture that was specifically designed to accommodate a maximum plate width of 250 mm (see Figure 4). To effectively extract the resulting chips in the form of dust, a powerful NEDERMAN extraction system (Nederman Holding AB, Helsingborg, Sweden) was used, along with a specially designed nozzle created via 3D printing. These methods were selected based on scientific articles [27,28]. The most common form of machining of CFRP materials is contour cutting of laminated parts; therefore, this study focused on the problems that arise in this method of machining.

**Figure 5:**
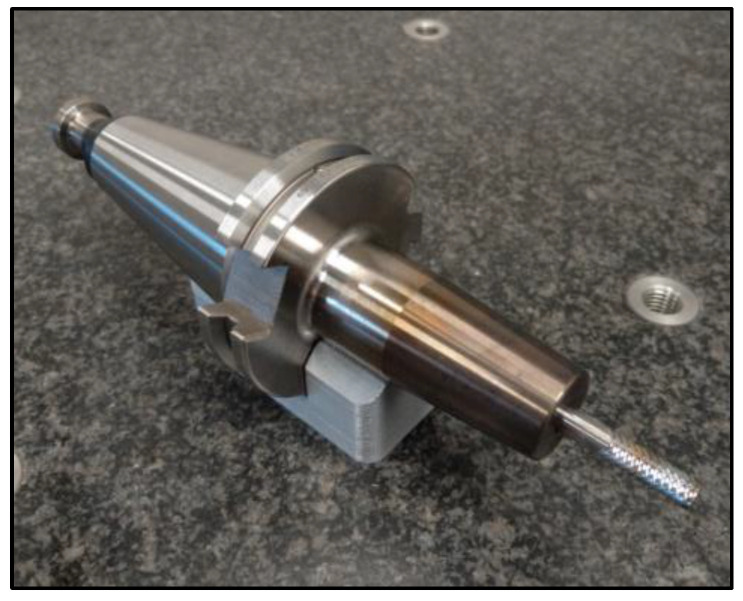
Clamped tool.

For the research, a specific type of monolithic 6 mm diameter uncoated sintered carbide cutter manufactured by UniCut s.r.o (Holoubkov, Czech Republic) was utilized. This cutter was specifically designed for the contour cutting of composite materials. The tool was securely clamped in a thermal chuck, which is known for its strong clamping force and low runout value of 0.003 mm (see Figure 5). The influence of the clearance angle on the observed accompanying and subsequent phenomena was investigated for the cutters. Three different clearance angles were chosen (8.4°, 12.4°, and 16.4°), and the rest of the tool geometry remained unchanged. The manufacturer of these tools was consulted, who recommended the clearance angle values. The clearance angle plays a significant role in machining composite materials. A larger clearance angle makes it more challenging for delaminated fibers to rub against the tool back and cause tool abrasion. However, a larger clearance angle also results in a lower blade angle, which reduces the stability of the blade. During the milling process, the generated forces and the following phenomena were monitored: tool wear, surface roughness of the milled edge of the workpiece, and delamination on the edge of the workpiece.

Cutting conditions for milling were chosen according to the tool manufacturer’s recommendations, as shown in Table 2.

Machining was performed on a three-axis milling center DMG MORI CMX 600 (DMG Mori Seiki, Nagoya, Japan) with a spindle power of 13 kW and a maximum speed of 12,000 rpm. Non-contiguous machining was selected to suppress delamination.

All selected parameters were measured after a certain time, as shown in Table 3.

The displacement component of the cutting force F_y_ was measured using a KISTLER 9265 B piezoelectric dynamometer (Kistler Instrument Corp, Amherst, NY, USA). The surface roughness of the machined edge of the material was measured using a MITUTOYO SV-2000N2 SURFTEST touch profile profilometer (Mitutoyo, Kanagawa, Japan), the evaluation of the measured profile was performed using Surfpak software (v.12.2, 2004, Mitutoyo, Kanagawa, Japan), and the evaluation was performed only for the parameter Ra, which is the most telling for machining composites. The wear, tooling, and size of the delaminated material fibers were investigated using a KEYENCE VK-X1100 3D laser confocal microscope (Keyence, Itasca, IL, USA). The microscope was used to evaluate the amount of wear on the back of the VB tool, and the size and type of the delaminated fibers were examined.

Statistical data processing involves calculating the arithmetic mean, denoted as ‘x’, from the measured data. Afterward, the measurement uncertainty was calculated.

The measurement uncertainty was calculated according to the valid document Guide to the Expression of Uncertainty in Measurement (document EA 4/02). First, the A-type uncertainty was determined, and then the B-type uncertainty, the combined standard uncertainty u(s), and the resulting expanded measurement uncertainty U were calculated. In this case, the standard uncertainties were the sample standard deviation of the mean based on the calculation. The procedure for determining the type B standard uncertainty was based on determining the uncertainty by means other than the statistical analysis of a series of observations. In this specific case, the standard uncertainty determination involved several factors. First, it relied on the information obtained from the calibration sheet of the measuring instrument. Additionally, the uncertainties associated with various influences acting on the measurement process were taken into account. These influences included, among others, the temperature coefficient of the length expansion of the cutting tool, temperature variations in the measuring room, and the influence of the operator handling the measuring instrument. The combined standard uncertainty was calculated as the geometric sum of the A-type uncertainty and the B-type uncertainty. This expanded uncertainty of measurement U was multiplied by the standard uncertainty u(y) of the estimate of the output quantity y by the expansion factor k: U = k × u(y). For the calculation, the normal (Gaussian) distribution of the measurand was determined (the standard uncertainty of the estimate of the output quantity was determined with sufficient reliability); therefore, the standard expansion coefficient k = 2 was used. The resulting expanded uncertainty corresponded to a coverage probability of approximately 95%. 

## 3. Results

To obtain the necessary results, it was necessary to evaluate the magnitudes of the cutting forces, tool wear, the surface roughness of the machined material, and the size of the delaminated layer on the edge of the machined material.

### 3.1. Tool Wear Assessment

**Figure 6:**
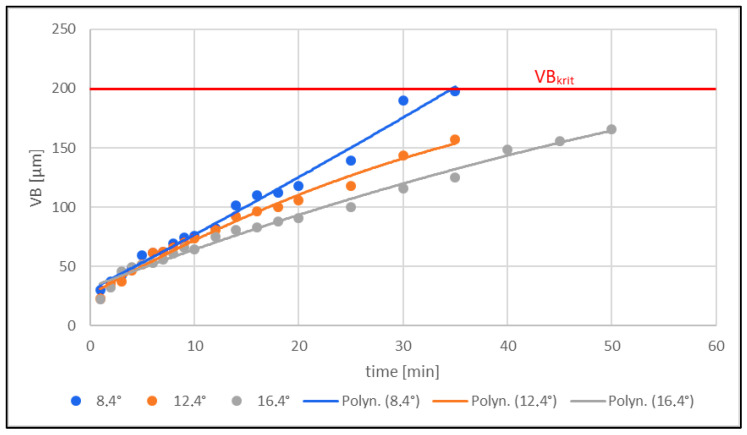
Effect of clearance angle value on tool wear VB [µm].

Wear measurements were taken at five selected locations around the circumference of the tool. At each selected location, the wear on the tool back was measured five times for each type of tool. The critical life of the VB_krit_ tool was determined according to the recommendations provided by the tool manufacturer. The dependence between the tool wear and milling time was investigated. Based on the measured results shown in Table 3 and graphically presented in Figure 6, the following conclusions can be drawn:

(1) Tool wear increased with increasing milling time.
(2) Tool wear increased with a lower tool clearance angle.

The VB wear [µm] of all three tools with different clearance angles (8.4°; 12.4°; 16.4°) increased with increasing milling time. The tool with a clearance angle of 8.4° showed faster back wear (the critical tool life of VB_krit_ = 200 µm was reached within t = 35 min) than the tool with a clearance angle of 16.4°, where the increase was rather gradual, and the critical tool life of VB_krit_ = 200 µm was not even reached in 50 min of milling. The wear on all three instruments increased with decreasing (smaller) clearance angle. The tool with a clearance angle of 8.4° had the highest wear (VB = 197.83 µm at the observed time t = 35 min), whereas the tool with the highest clearance angle of 16.4° had the lowest wear (VB = 124.80 µm at the observed time).

### 3.2. Roughness Evaluation of the Machined Edge/Surface

**Figure 7:**
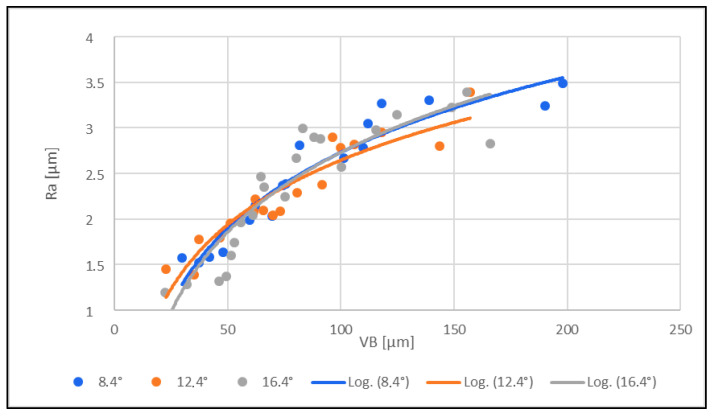
Effect of the machined edge roughness Ra [µm] on tool wear VB [µm].

The dependence between the roughness of the machined edge and the tool wear was investigated. Ra [µm] was selected as the parameter for the study of this dependence. The surface roughness was measured using a MITUTOYO SV-2000 contact profilometer with a 5 µm radius diamond tip. The measured profile was evaluated according to the applicable standard, ČSN ISO 21920 [29]. The measured results are presented in Figure 7.

From the measured results graphically presented in Figure 7, the following conclusions can be drawn:

(1) Tool wear VB [µm] affected the surface roughness for all three tools with different clearance angles (8.4°; 12.4°; 16.4°). The roughness Ra of the machined edge increased with increasing tool wear.
(2) The roughness of the machined edge and the tool wear did not increase or decrease significantly with a greater or lesser angle of the tool back.

### 3.3. Cutting Force Rating

**Figure 8:**
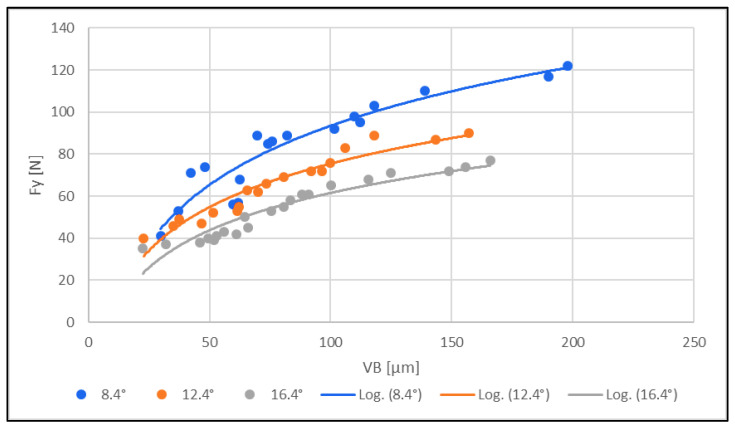
Effect of cutting force F_y_ [N] on tool wear VB [µm].

Furthermore, the relationship between the cutting force (sliding cutting force F_y_) and the tool wear was investigated. The measured results are shown in Figure 8.

From the measured results graphically presented in Figure 8, the following conclusions can be drawn:

(1) The cutting force (sliding) F_y_ [N] increased with increasing tool wear for all three tools with different clearance angles (8.4°; 12.4°; 16.4°).
(2) The cutting force for all three instruments increased with a lower clearance angle.

**Figure 9:**
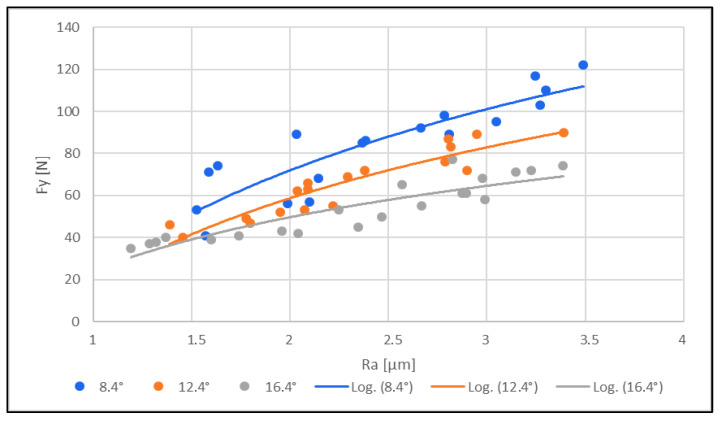
Effect of cutting force F [N] on the roughness of the machined edge Ra [µm].

The relationship between the cutting force (sliding cutting force F_y_) and the roughness of the machined edge was also investigated. The measured results are shown in Figure 9.

### 3.4. Evaluation of Delamination

**Figure 10:**
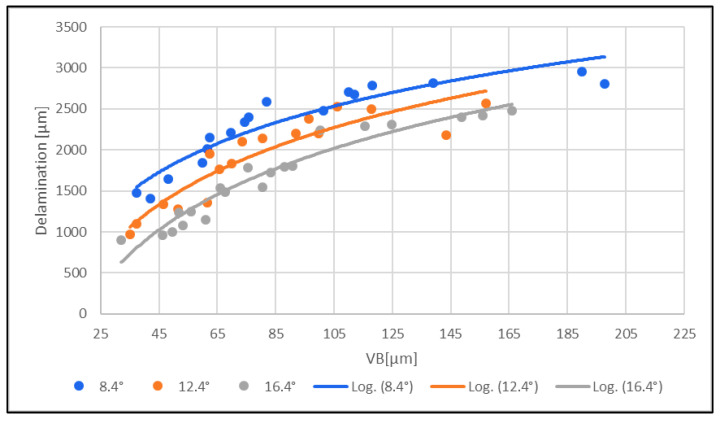
Effect of delamination [µm] on the top edge of the machined surface on tool wear VB [µm].

**Figure 11:**
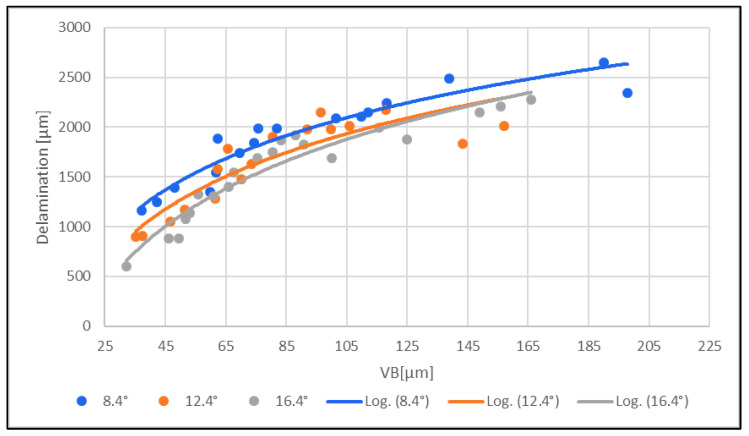
Effect of delamination [µm] on the bottom edge of the machined surface on tool wear VB [µm].

**Figure 12:**
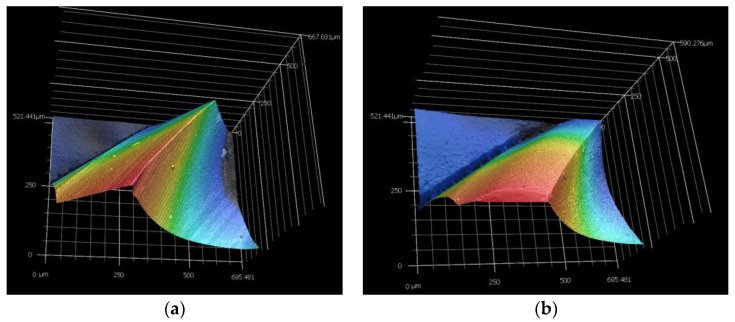
Detail of the tool edge: (**a**) new blade; (**b**) worn blade.

The dependence of the clearance angle and tool wear on the size of delamination was investigated. The measured results are graphically presented in Figure 10 and Figure 11. The clearance angle influences the formation and length of the delaminated fibers. A larger clearance angle resulted in a smaller size of delaminated fibers. Additionally, the size of the delaminated fibers increased with increasing wear. Furthermore, the radius of curvature of the blade increased with more wear on the tool back (unworn blade, Figure 12a; worn blade, Figure 12b). This indicates that the blade became blunted and lost its ability to cut the individual carbon fibers of the top layer of the CFRP material.

(1) Delamination [µm] increased with increasing tool wear for all three tools with different clearance angles (8.4°; 12.4°; 16.4°).
(2) Delamination and tool wear for all three tools increased with a lower clearance angle.

The delamination [µm] increased with increasing tool wear for all three tools with different clearance angles (8.4°; 12.4°; 16.4°).

Within the conducted measurements, attention was also given to the type of delamination that primarily occurred on the upper side of the machined CFRP edge. 

**Figure 13:**
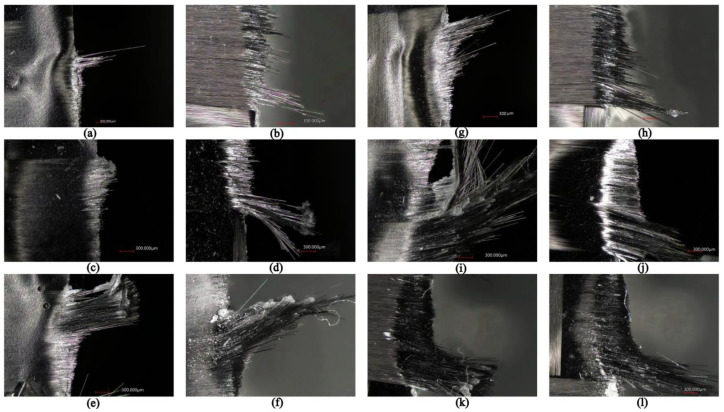
Delamination after milling time (**a**,**b**) 8.4° (10 min), top edge of the machined surface; (**c**,**d**) 12.4° (10 min), top edge of the machined surface; (**e**,**f**) 16.4° (10 min), top edge of the machined surface; (**g**,**h**) 8.4° (35 min), bottom edge of the machined surface; (**i**,**j**) 12.4° (35 min), bottom edge of the machined surface; (**k**,**l**) 16.4° (35 min), bottom edge of the machined surface.

Two types of delaminations were observed as follows: type I/II and type III. The delamination of type I/II was most noticeable at the lower machined edge and was characterized by a significant presence of uncut fibers that protruded and were either bent by the advancing tool in the cutting direction or only slightly trimmed (see Figure 13a–c). The poorest quality machined edge, indicated by uncut protruding fibers, was observed after a machining time of t = 35 min, demonstrating the significant influence of tool wear on delamination (Figure 13g–l). It was observed that a sharp tool resulted in clean fiber cuts with no delamination, whereas a tool with average wear already showed visible uncut protruding fibers. As the wear increased, pronounced delamination occurred, including uncut protruding fibers and fiber breakage within the individual layers of the milled CFRP edge. Additionally, clusters of crushed epoxy resin were observed on the surface of the machined edge, forming a fine crust on the uncut fibers and along the machined edge.

Occasionally, type III delamination was also observed. Type III delamination was presented as loose fibers that partially adhered to the machined surface and were oriented parallel to the tool feed direction. These loose fibers resulted in a poor quality ‘unsharp’ machined surface, reminiscent of the burrs commonly encountered in machining metallic materials. This type of delamination primarily occurred at the upper edge of the machined surface.

## 4. Discussion

This study investigated the effect of the clearance angle of a milling tool on the wear, forces, roughness of the machined edge, and delamination of a non-consistently milled CFRP board with a twill weave and 90° fiber orientation.

Tool wear VB [µm] increased with increasing milling time for all three tools with different clearance angles (8.4°; 12.4°; 16.4°). Furthermore, tool wear increased with a lower clearance angle (8.4°).

It was also observed that the smooth and shiny surface in the tool wear area indicated the presence of abrasion wear. This abrasion wear was caused by the high abrasiveness of the carbon fibers. The chips that rubbed against the tool’s back during the machining process acted as a polishing mechanism, resulting in a shiny and polished area in the tool wear zone. However, unlike the wave-like wear observed in [27,28], the chips did not produce wave-shaped wear patterns. This difference could be attributed to the distinct geometry of the examined tools, which featured numerous small teeth with opposite helix angle orientations.

The overall measurements show that the tool geometry, or the clearance angle, significantly influenced the tool wear during the milling process. The larger the tool clearance angle (16.4°), the less the wear; conversely, the smaller the tool clearance angle (8.4°), the greater the wear.

Ra [µm] was chosen as a parameter for the dependence study. The roughness of the machined edge increased with increasing tool wear for all three tools with different clearance angles (8.4°; 12.4°; 16.4°). Further, the roughness of the machined edge and tool wear did not increase or decrease significantly with smaller or larger tool clearance angles. The observed effect was not demonstrated in any way. Also, in [30], changing the clearance angle did not affect the amount of surface roughness.

The overall measurements showed that the tool geometry, or the clearance angle, did not significantly affect the worn tool’s effect on the machined edge’s roughness.

The cutting force F_y_ [N] increased with increasing tool wear for all three tools with different clearance angles (8.4°; 12.4°; 16.4°). Further, the cutting force and tool wear increased with a lower tool clearance angle (8.4°).

The overall measurement subsequently showed that the cutting (sliding) force increased significantly with increasing wear. Both the cutting force and tool wear decreased with increasing tool clearance angle. With a low tool clearance angle (8.4°), both the cutting force and tool wear increased. A low cutting force prevented tool wear.

The small magnitude of the cutting forces resulted in low tool wear, and thus low energy consumption.

The cutting force F_y_ [N] for all three tools with different clearance angles (8.4°; 12.4°; 16.4°) increased with increasing roughness of the machined edge. Furthermore, the cutting force and roughness of the machined edge increased with a lower tool clearance angle (8.4°).

The overall measurements showed that the cutting (sliding) force increased significantly with increasing surface roughness of the machined edge. Both the cutting force and surface roughness decreased with increasing tool clearance angle (16.4°). With a low tool clearance angle (8.4°), both the cutting force and surface roughness increased. The overall low cutting force helped to reduce surface roughness and prevent tool wear.

Delamination [µm] increased with increasing roughness of the machined edge, both on the upper and lower sides of the edge, for all three tools with different clearance angles (8.4°; 12.4°; 16.4°). Furthermore, the delamination and roughness of the machined edge increased with a lower tool clearance angle (8.4°). The resulting delamination, in the form of fibers protruding beyond the edge of the material, was the main type of delamination observed, in contrast to [6], where the delamination was mainly in the form of deep abrasion of the overlying material

The overall measurements subsequently showed that delamination increased significantly with an increase in the surface roughness of the machined edge. Both the delamination and surface roughness decreased with increasing tool clearance angle (16.4°). With a low tool clearance angle (8.4°), both delamination and surface roughness increased.

It was observed that delamination always occurred at the point of initial contact between the tool and the CFRP board fiber where the initial cutting occurred. The fibers were under compressive stress at the point of contact with the tool, and cracks started to form, with tension and subsequent fracture of the fibers on the opposite side. The polymer matrix was stressed by the pressure in front of the tool, cracks formed, and the brittle matrix was crushed into small particles.

Delamination [µm] increased with increasing tool wear for all three tools with different clearance angles (8.4°; 12.4°; 16.4°), both on the upper and lower sides of the machined edge. Furthermore, delamination and tool wear increased with a lower tool clearance angle (8.4°).

The above results show that delamination increased with increasing tool wear. Both delamination and tool wear decreased with increasing tool clearance angle (16.4°). With a low tool clearance angle (8.4°), both delamination and tool wear increased.

Delamination [µm] increased with increasing cutting force for all three tools with different clearance angles (8.4°; 12.4°; 16.4°), both on the upper and lower sides of the machined edge. Increasing the cutting force increased the degree of delamination. Furthermore, delamination and cutting force increased with a lower tool clearance angle (8.4°).

The above results show that delamination increased with increasing cutting force. Both delamination and cutting force decreased with increasing tool clearance angle (16.4°). With a low clearance angle (8.4°) of the tool, both delamination and cutting force increased. The formation of delamination is consistent with the data in [31].

## 5. Conclusions

Tool geometry has a significant influence when machining CFRP materials. This study focused on the investigation of the effect of the tool clearance angle on wear, cutting forces, machined edge roughness, and delamination in the non-contiguous milling of CFRP laminates.

The following conclusions were drawn from the measurements:

- The angle of the tool back has a significant effect on tool wear, the roughness of the machined edge, and the delamination of CFRP.
- The tool with a clearance angle of 8.4° wore out faster than the tool with a clearance angle of 16,4°. The same was true for the cutting force, the observed roughness of the machined edge, and delamination. In this context, it was shown that the cutting force increased with increasing tool wear, which in turn increased surface roughness and delamination.
- From the tool surface topography images, it was observed that abrasion wear occurred within the tool wear.
- Type I/II and III delaminations were observed in the machined upper and lower edges. Type I/II delamination was the most pronounced and increased with increasing tool wear, especially on the lower machined edge.

The results of this research hold significant practical importance, not only in terms of the quality of the machined surface but also in relation to time, financial costs, and energy savings when machining CFRP composite materials. Another possible direction for future research could be to investigate other aspects of tool geometry, such as face angle, helix pitch angle, etc., and their influence on the parameters investigated in the present research.

## Figures and Tables

**Table 1:** Properties of the machined materials.

Resin Type	Epoxy
Fiber	Hyosung Tansome 12K H2550
Weave	Twill 2/2
Areal weight	600 g/m^2^ ± 3%
Number of filaments per roving	3K
Ply thickness in laminate	4.3 mm

**Table 2:** Cutting conditions.

Parameter	Value
Diameter of the tool	6
Cutting speed v_c_	220 m/min
Feed rate vf	1167 mm/min
Sidestep a_e_	1 mm

**Table 3:** Effect of tool wear on milling time.

Tool	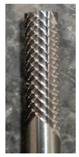 8.4°	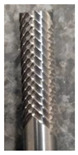 12.4°	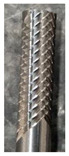 16.4°
time t [min]	wear VB [µm] ± measurement uncertainty U [µm]		
10	75.77 ± 0.89	73.43 ± 0.88	64.57 ± 0.99
20	118.09 ± 0.88	106.01 ± 0.87	90.91 ± 0.95
35	197.83 ± 0.95	157.09 ± 0.89	124.8 ± 0.98
50	-	-	165.88 ± 0.99

## Data Availability

Data sharing does not apply to this article.
